# A New Modified Histogram Matching Normalization for Time Series Microarray Analysis

**DOI:** 10.3390/microarrays3030203

**Published:** 2014-07-01

**Authors:** Laura Astola, Jaap Molenaar

**Affiliations:** 1 Department of Biomedical Engineering, Eindhoven University of Technology, Eindhoven 5612 AZ, The Netherlands; 2 Biometris, Wageningen University and Research Centre, Wageningen 6708 PB, The Netherlands; E-Mail: jaap.molenaar@wur.nl; 3 Wageningen Centre for Systems Biology, Wageningen 6700 AC, The Netherlands

**Keywords:** quantile normalization, histogram matching, time series

## Abstract

Microarray data is often utilized in inferring regulatory networks. Quantile normalization (QN) is a popular method to reduce array-to-array variation. We show that in the context of time series measurements QN may not be the best choice for this task, especially not if the inference is based on continuous time ODE model. We propose an alternative normalization method that is better suited for network inference from time series data.

## 1. Introduction

When studying temporal changes in gene expression levels via microarrays, it is important to reduce any systematic biases, since one should only compare measurements on an equal footing. Normalization is the term used for the varying techniques applied to microarray data to achieve this reduction. Several approaches have been proposed and quite thoroughly investigated in the past [1,2,3,4]. The strongest effect in terms of time series analysis arise from inter-array normalization procedures, since each array represents a time point. Therefore, in this paper we focus only on inter-array normalization.

Given microarray time series data, one may try to unravel the structure of an underlying gene network by constructing a mathematical/computational model and to test whether this can reproduce the observations. If this is the case such a model can be very useful for experiments *in silico* and assist in designing experiments. Popular models include graphical models [5,6,7,8] and systems of ordinary differential equations (ODEs) [9,10,11,12]. The usefulness of ODEs is underlined by the fact that they can be used to realistically simulate the dynamics of gene expressions [13,14]. We consider the effect of normalization on these models by first generating artificial data with a known network and by investigating how well we can reconstruct the original network after normalization is applied to the data. For this we compare the performances of two different normalization methods: the often employed quantile normalization (QN) and a newly developed “modified histogram matching normalization (MHMN)”. In addition we apply both algorithms also to real large scale microarray data. In the following section, we present our algorithm in detail and in the results section we discuss the results of our experiments. A Mathematica code that performs the new normalization is provided on request. In the Appendix we show both normalization procedures using a simple toy example data.

## 2. Methods

### Algorithm for Modified Histogram Matching Normalization

In this section we describe our new algorithm, which we refer to as modified histogram matching normalization. We assume that the expression data have dimensions M×N, *i.e.*, *M* genes and *N* time points. For convenience we illustrate the procedure step by step in the Appendix. To normalize the data, we proceed as follows:

(1) First sort all data in the whole data matrix according to magnitude from low to high;
(2) Partition this sorted dataset into bins B(i) (i=1,…,M), each bin containing exactly *N* numbers;
(3) Sort each column in the original unsorted data matrix according to magnitude from low to high. This results in an M×N matrix *S* with elements $s_{ij}$, where each column contains the same elements as in the original unsorted data matrix but in an order where the smallest values are on top and largest at the bottom;
(4) For i=1,…,M and j=1,…,N, scale all elements in the *i*th row of matrix *S* using the following scaling function *f*
  (1) $$f\left(s_{ij}\right)=\left(\max(B(i))-\min(B(i))\right)\frac{s_{ij}-\min\left(S\left(i\right)\right)}{\max(S(i))-\min(S(i))}+\min\left(B\left(i\right)\right)\;$$
  so that the first (smallest) element $f(s_{i1})$ equals the first value in the interval B(i) and the last element $f(s_{iN})$ equals the last value of the bin B(i);
(5) Return each scaled element in each column back to their original unsorted positions within the columns.

The difference with QN is that instead of averaging over the rows of column-wise sorted data, these expression levels are scaled to fit the appropriate bins in the histogram of the distribution of expression levels over time. In this way the variation present in the original data is preserved. This is important especially when doing network inference based on a continuous ODE-model that relates the rate of change of an expression level of one gene to the expression levels of other (regulating) genes. For a discrete Boolean-type network inference, QN is accurate enough, because it preserves the rank information. However, for a ODE-based inference, which is essentially continuous time approach, it is crucial to have more than merely the rank information.

## 3. Results

As mentioned in the introduction, time series data are often analyzed using graphical models or systems of ODEs. To not lose our focus because of technical details, we use here the simplest representative of graphical models, namely correlation. In spite of its simplicity, it is often a very useful tool to give an impression of expression level data and the potential connections between the genes involved. We investigate, both with simulated and real microarray data, how the correlations deviate from the “ground truth” after applying QN and MHMN normalization.

In the subsequent analysis, we investigate how well QN and MHMN allow reconstruction of the parameters in linear ODEs. The latter are the simplest representatives of ODEs. Because the temporal dynamics of gene network are often simulated using ODEs [10,12,15], this is a critical test. Since knowing the parameters in the ODEs corresponds to knowing the interactions between genes, it is important that the estimation of parameters is not blurred by normalization.

### 3.1. Effects on Correlation

**Figure 1:**
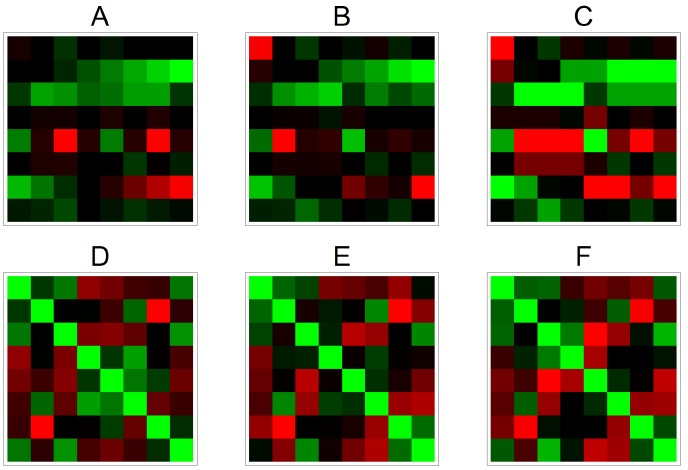
On the top row (**B** and **C**) we have heat maps of the original data (**A**) and of the same data after applying HMHN and QN respectively. The second row (**D**,**E**,**F**) shows the heat maps of the correlations of the data in the first row. Thus, the color of a block in *i*th row and *j*th column of the matrix below indicates how strongly the rows *i* and *j* in the matrix above are correlated.

**Figure 2:**
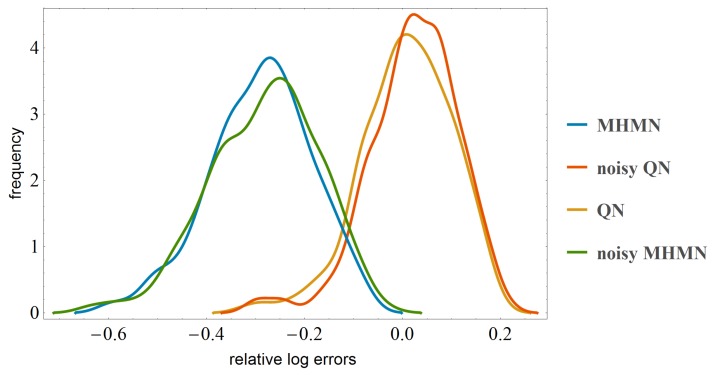
Distributions of mean log errors in correlations after QN and MHMN normalization. These distributions were obtained from 500 simulations.

We want to see how the correlations of time series measurements are affected by the two different normalization methods. To give an impression of the results, we take as example a dataset with 8 genes and 8 time points, stored in an 8×8 array shown in Figure 1. From Figure 1, we may conclude that MHMN normalization preserves the correlation structure of the data matrix somewhat better than QN for this particular case. To obtain a more quantitative measure on the performance of MHMN and QN, we need to test them on a variety of datasets. To simulate time series data, we have generated 500 random time series data with expression levels of 8 genes and 16 time points. Three genes show clear temporal patterns, which are increasing, decreasing, and sinusoidal. The other five genes have random values of varying levels. Then we apply to each dataset a random plate-effect by choosing randomly one time point and adding a random amount of additive noise to the data at this time point. Next, we apply the two normalizations to both noiseless and noisy data. Finally we compare the correlation matrices of the results with the correlation matrices of the original data. As a measure to capture the differences between matrices, we use a popular matrix norm, the Frobenius norm. From the simulation results shown in Figure 2, it is immediate that MHMN results in significantly more accurate estimates than QN.

### 3.2. Effects on Correlation on Real Data

**Figure 3:**
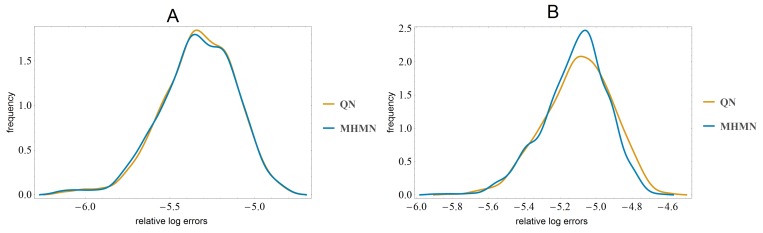
In panel (**A**), we have plotted the smoothed histograms of average errors, when there is no plate-specific noise. In panel (**B**), similarly, but time-point specific additive noise applied to the data.

The real microarray data we employ in this section is the time series data generated by Sokolović *et al.* in their study on the effects of fasting on murine transcriptomics [16] in various organs including the liver and the brain. We used the data that consists of expression levels in brain tissue of *Mus musculus* at 5 time points after 0, 12, 24, 36, and 72 h of fasting. Data has dimensions 22,680×5 and the correlation matrices are of size 22,680×22,680 . In network inference, one typically is interested in some subset of genes instead of the total set of genes. By focusing on a small subset one can formulate hypotheses that are experimentally easier to test. To simulate small network inference, we randomly choose sample sets of ten genes and compare their correlations before and after normalization with and without time-point specific noise. Similarly to the simulated experiments in the previous section, we plotted the histograms of Frobenius norms of errors generated by 1000 samples in Figure 3.

We observe that indeed when the data is very large, the errors in correlations have similar distributions for QN and MHMN. According to the Kolmogorov–Smirnov test, the null hypothesis that the datasets have the same distribution at the 99% confidence level is not rejected. Also, when we compare the correlations of the total set of genes, the total residuals in correlations are identical up to three significant digits. This suggests that in the case of very large datasets MHMN performs similarly to QN.

### 3.3. Effects on Reverse-Engineering via ODEs

**Figure 4:**
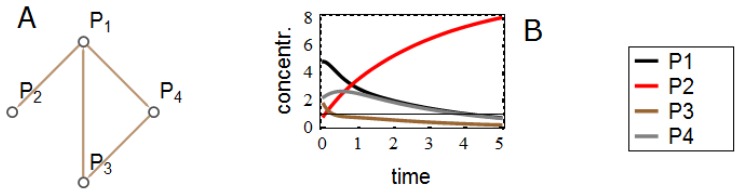
(**A**) Example of a network structure with 4 nodes and 4 interactions. (**B**) Solutions of the ODE for $x=(P_{1},P_{2},P_{3},P_{4})$.

In this section we simulate time series data using linear ODEs. First we generate random gene networks with a fixed number of genes and varying number of edges. Such a network correspond to a binary adjacency matrix, where a matrix element (i,j) (*i* = row number, *j* = column number) has value 1 if gene *j* is connected to gene *i* and zero otherwise. With this same adjacency matrix, we can set up a system of linear ODEs by replacing all ones with appropriate random numbers to model the strength of interaction/regulation from gene *j* to gene *i*. The resulting matrix *M* is used in the linear ODE system $\frac{dx}{dt}=Mx$, with *x* the vector containing the gene level expressions. Finally we choose a set of random initial values within a reasonable range. By integrating the ODE we obtain continuous time solutions x(t). We sample the obtained solutions with regular intervals to obtain time series data. An example network and the corresponding simulated data are shown in Figure 4.

The task is now to infer the entries in matrix *M* from these samples, first using the samples directly, second, using the samples after applying MHMN and QN-normalization respectively. To simulate a plate effect, we put additive noise to the samples at a randomly chosen time point.

**Figure 5:**
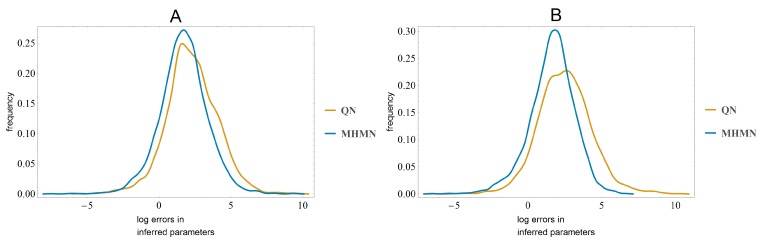
In panel (**A**) we use noiseless data and compare the inferred parameters after QN and MHMN normalizations in each experiment to the original parameters used in generating data. In panel (**B**) the data contains time-point specific multiplicative noise. In both cases QN gives significantly larger errors than MHMN.

We performed series of simulation experiments with small systems of ODEs (4–5 nodes). With or without time-point specific noise (plate effect), MHMN consistently resulted in better parameter inference than QN. In each experiment a random set of max 25 parameters need to be inferred. In both cases the Kolmogorov–Smirnov test leads to rejecting the null hypothesis that the datasets have the same distribution at the 99% confidence level. In Figure 5 we show the distributions of log errors in the inferred parameters.

## 4. Conclusions

We have shown that the popular normalization method, quantile normalization (QN), while being effective in equalizing the data from array to array, was in our analysis inferior to MHMN for ODE-based time series analysis. It is also less suitable for correlation-based analysis, when the number of rows in the data is not large. Time series analysis is important in practice. For example, when we consider tissues of a developing organism, one expects to see temporally meaningful changes in expression levels. QN is less suitable for time series analysis because it loses the information on the differences of expression levels between arrays. QN produces arrays that consist of exactly the same numerical values in each column (time point). See Appendix for an illustration. The modified histogram matching normalization (MHMN) that we propose here does in some sense the opposite. After the initial histogram matching, where each array has a distribution of the whole dataset, within each bin the values are scaled to preserve the contrast between different expression values. The largest difference between the two methods we see in the parameter estimation task, when using an ODE-model. Parameter inference is crucial in determining both the topology of the network and the nature of interactions in the network. Based on our simulation studies, we recommend MHMN rather than QN for ODE-based network inference using time series microarray data that is susceptible for array-to-array noise.

## Appendix

We illustrate the normalization procedures step by step using a 3 by 3 matrix *M*. Matrix *M* represents a microarray with 3 genes and 3 time measurements. Each row corresponds to a gene expression level and each column corresponds to a time point. Matrix *I* keeps track of the position of the elements in the matrix

(2) $$M=\left(\begin{array}{ccc} 5 & 5 & 5\\ 3 & 4 & 4\\ 2 & 4 & 6 \end{array}\right)\;I=\left(\begin{array}{ccc} \left(1,1\right) & \left(1,2\right) & \left(1,3\right)\\ \left(2,1\right) & \left(2,2\right) & \left(2,3\right)\\ \left(3,1\right) & \left(3,2\right) & \left(3,3\right) \end{array}\right)$$

## Quantile normalization

(1) First each column is ordered so that the smallest value comes to the top:
  (3) $$M^{\prime }=\left(\begin{array}{ccc} 2 & 4 & 4\\ 3 & 4 & 5\\ 5 & 5 & 6 \end{array}\right)\;I^{\prime }=\left(\begin{array}{ccc} \left(3,1\right) & \left(2,2\right) & \left(2,3\right)\\ \left(2,1\right) & \left(3,2\right) & \left(1,3\right)\\ \left(1,1\right) & \left(1,2\right) & \left(3,3\right) \end{array}\right)\;$$
(2) Then each value is replaced by the row mean. For example the row mean for the first row is $\frac{1}{3}\left(2+4+4\right)=3.33\ldots$.
  (4) $$M^{\prime \prime }=\left(\begin{array}{ccc} 3.33 & 3.33 & 3.33\\ 4 & 4 & 4\\ 5.33 & 5.33 & 5.33 \end{array}\right)\;I^{\prime }=\left(\begin{array}{ccc} \left(3,1\right) & \left(2,2\right) & \left(2,3\right)\\ \left(2,1\right) & \left(3,2\right) & \left(1,3\right)\\ \left(1,1\right) & \left(1,2\right) & \left(3,3\right) \end{array}\right)\;$$
(3) Finally each element is returned to their original position:
  (5) $$QN\left(M\right)=\left(\begin{array}{ccc} 5.33 & 5.33 & 4\\ 4 & 3.33 & 3.33\\ 3.33 & 4 & 5.33 \end{array}\right)\;I=\left(\begin{array}{ccc} \left(1,1\right) & \left(1,2\right) & \left(1,3\right)\\ \left(2,1\right) & \left(2,2\right) & \left(2,3\right)\\ \left(3,1\right) & \left(3,2\right) & \left(3,3\right) \end{array}\right)\;$$

## Modified histogram matching normalization

In MHMN, the first step ($M^{\prime }$) is the same as in QN.

(2) Data is ordered and divided into bins: B(1)=(2,3,4), B(2)=(4,4,5) and B(3)=(5,5,6).
(3) Instead of taking the row means, the scaling function *f* as defined in Section 2 is applied to each element. For example element $M^{\prime }\left(2,1\right)=3$ and
  (6) $$\begin{array}{cc} f(3) & =\left(\max(B(2))-\min(B(2))\right)\frac{\left(3-\min(S(2))\right)}{\left(\max(S(2))-\min(S(2))\right)}+\min\left(B\left(2\right)\right)\\ & =\left(5-4\right)\frac{\left(3-3\right)}{\left(5-3\right)}+4=4\; \end{array}$$
  This scaling results in the following matrix:
  (7) $$M^{\prime \prime }=\left(\begin{array}{ccc} 2 & 4 & 4\\ 4 & 4.5 & 5\\ 5 & 5 & 6 \end{array}\right)\;I^{\prime }=\left(\begin{array}{ccc} \left(3,1\right) & \left(2,2\right) & \left(2,3\right)\\ \left(2,1\right) & \left(3,2\right) & \left(1,3\right)\\ \left(1,1\right) & \left(1,2\right) & \left(3,3\right) \end{array}\right)\;$$
(4) Finally the scaled elements are returned into their original positions:
  (8) $$MHMN\left(M\right)=\left(\begin{array}{ccc} 5 & 5 & 5\\ 4 & 4 & 4\\ 2 & 4.5 & 6 \end{array}\right)\;I=\left(\begin{array}{ccc} \left(1,1\right) & \left(1,2\right) & \left(1,3\right)\\ \left(2,1\right) & \left(2,2\right) & \left(2,3\right)\\ \left(3,1\right) & \left(3,2\right) & \left(3,3\right) \end{array}\right)\;$$
